# DNA Detection Using Recombination Proteins

**DOI:** 10.1371/journal.pbio.0040204

**Published:** 2006-06-13

**Authors:** Olaf Piepenburg, Colin H Williams, Derek L Stemple, Niall A Armes

**Affiliations:** **1**ASM Scientific Ltd, Cambridge, United Kingdom; **2**Wellcome Trust Sanger Institute, Hinxton, Cambridge, United Kingdom; Brandeis UniversityUnited States of America

## Abstract

DNA amplification is essential to most nucleic acid testing strategies, but established techniques require sophisticated equipment or complex experimental procedures, and their uptake outside specialised laboratories has been limited. Our novel approach, recombinase polymerase amplification (RPA), couples isothermal recombinase-driven primer targeting of template material with strand-displacement DNA synthesis. It achieves exponential amplification with no need for pretreatment of sample DNA. Reactions are sensitive, specific, and rapid and operate at constant low temperature. We have also developed a probe-based detection system. Key aspects of the combined RPA amplification/detection process are illustrated by a test for the pathogen methicillin-resistant Staphylococcus aureus. The technology proves to be sensitive to fewer than ten copies of genomic DNA. Furthermore, products can be detected in a simple sandwich assay, thereby establishing an instrument-free DNA testing system. This unique combination of properties is a significant advance in the development of portable and widely accessible nucleic acid–based tests.

## Abstract 2

The authors describe a new technology for the amplification of target DNA that does not require thermocycling, which could find applications in detection of pathogens or bioterror agents in the field.

## Introduction

The amplification of DNA is an essential step in most nucleic acid–based testing strategies. Established amplification techniques rely on sophisticated instrumentation, such as temperature-regulating equipment or complex sample-handling procedures. While unproblematic for specialised laboratories, these requirements have hampered the uptake of nucleic acid analyses in point-of-use and field settings. The technology presented in this study, recombinase polymerase amplification (RPA), overcomes the technical difficulties posed by current DNA amplification methods. It does not require thermal denaturation of template and operates at a low and constant temperature. In combination with a novel probe-based detection approach, RPA constitutes a significant advance in the development of portable and widely accessible nucleic acid–based tests.

**Figure 1:**
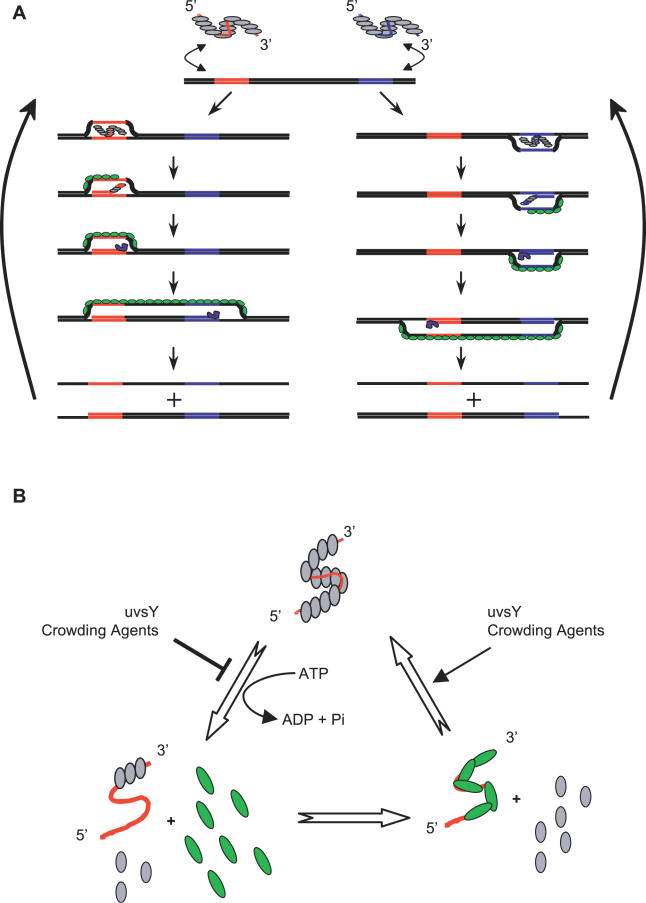
Schematic of the RPA Process and the Recombinase/Primer Filament Formation (A) Recombinase/primer filaments scan template DNA for homologous sequences (red/blue). Following strand exchange, the displaced strand is bound by gp32 (green), primers are extended by Bsu polymerase (blue). The net result of two opposing primer binding/extension events is the generation of one complete copy of the amplicon in addition to the original template. Repetition of the process results in exponential DNA amplification. (B) In the presence of ATP, uvsX (gray) binds cooperatively to oligonucleotides (red, top). Upon ATP hydrolysis, the nucleoprotein complex disassembles (left) and uvsX can be replaced by gp32 (green, right). The presence of uvsY and Carbowax20M shifts the equilibrium in favour of recombinase loading.

In RPA, the isothermal amplification of specific DNA fragments is achieved by the binding of opposing oligonucleotide primers to template DNA and their extension by a DNA polymerase ( Figure 1A). Global melting of the template is not required for the primers to be directed to their complementary target sequences. Instead, RPA employs recombinase-primer complexes to scan double-stranded DNA and facilitate strand exchange at cognate sites [ 1– 3]. The resulting structures are stabilised by single-stranded DNA binding proteins interacting with the displaced template strand, thus preventing the ejection of the primer by branch migration [ 4]. Recombinase disassembly leaves the 3′-end of the oligonucleotide accessible to a strand displacing DNA polymerase, in this case the large fragment of Bacillus subtilis Pol I (Bsu, [ 5]), and primer extension ensues. Exponential amplification is accomplished by the cyclic repetition of this process. The key to RPA is the establishment of a dynamic reaction environment that balances the formation and disassembly of recombinase-primer filaments ( Figure 1B). The recombinase we have employed, T4 uvsX, binds cooperatively to oligonucleotides in the presence of ATP. The resulting nucleoprotein complex actively hydrolyses ATP, and spontaneous recombinase disassembly in the ADP-bound state can lead to its replacement by T4 gp32, a single-stranded DNA binding protein necessary for the reaction. We found that a unique combination of T4 uvsY, a recombinase loading factor [ 6], and a particular crowding agent (Carbowax20M) establishes favourable reaction conditions that support RPA (see Protocol S1 and Figures S1 to S3). The proteins used in our approach are central components of in vivo processes required for cellular DNA synthesis, recombination, and repair and have been the subject of intensive research for a number of years [ 7]. In addition to facilitating DNA amplification in the RPA context, the dynamic reaction environment described here provides a system for the in vitro study of the recombination machinery and will aid the development of laboratory procedures that replace conventional hybridisation techniques.

## Results/Discussion

**Figure 2:**
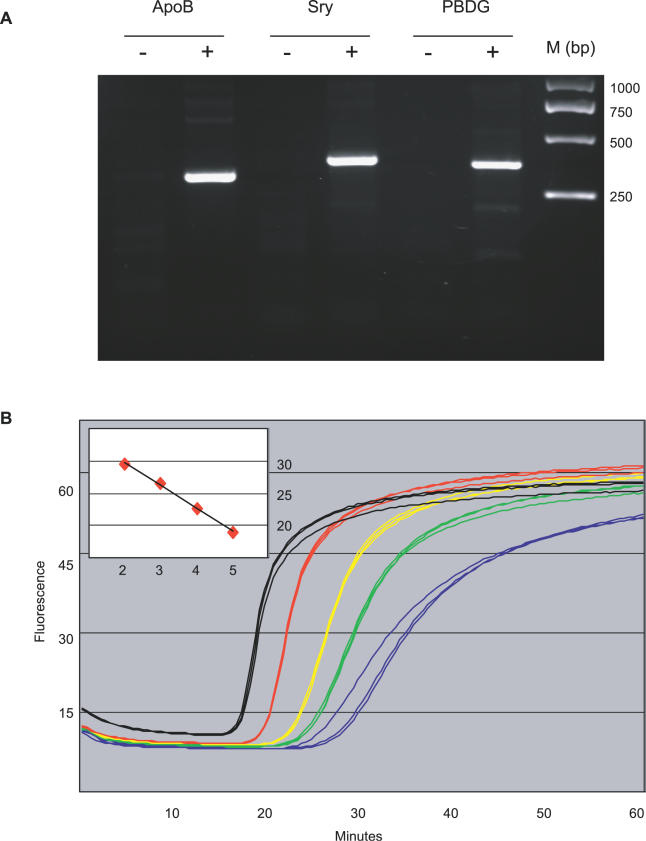
RPA Amplifies Specific Target Regions from Complex DNA Templates in less than 30 Minutes (A) Acrylamide gel electrophoresis of RPA products using primers for three human markers (apoB, Sry, PBDG). Water (−) or 1,000 copies of genomic DNA (+) served as template. Ten percent of each reaction is loaded on the gel. The expected product sizes are 305, 371, and 353 base-pairs for apoB, Sry, and PBDG, respectively. (B) Real-time RPA using primers for the B. subtilis SpoB locus. Fluorescence upon intercalation of SybrGreenI into nascent product is detected. B. subtilis DNA served as template in triplicate reactions at 10 ^5^ (black), 10 ^4^ (red), 10 ^3^ (yellow), or 100 copies (green) or water (blue). The onset of amplification depends linearly on the logarithm of the starting template copy number [see inset; time in minutes (midpoint of growth curve) versus log {template concentration in copy number)].

We applied the RPA process to a wide variety of targets in complex DNA templates. The versatility and specificity of the technology are exemplified by the amplification of three genetic markers, apolipoprotein B (apoB), sex-determining region Y (Sry), and porphobilinogen deaminase (PBDG), from complex human genomic DNA ( Figure 2A). While the negative controls did not produce a signal, clean amplification products of the correct identity ( Figure S4) were generated in each template-containing sample.

The progression of RPA reactions can be monitored in real-time by the inclusion of a sensitive nucleic acid dye ( Figure 2B) [ 8]. Here, primers for a locus in the B. subtilis genome have been used. The amplification of DNA proved to be exponential over a wide range of template concentrations and results were obtained in less than 30 min. The onset of amplification depends linearly on the logarithm of the starting number of template copies. Reactions carried out in the absence of template or at low template concentrations eventually generated a nonspecific signal, an effect brought about by a primer-dependent artefact ( Figure 2B, water control).

**Figure 3:**
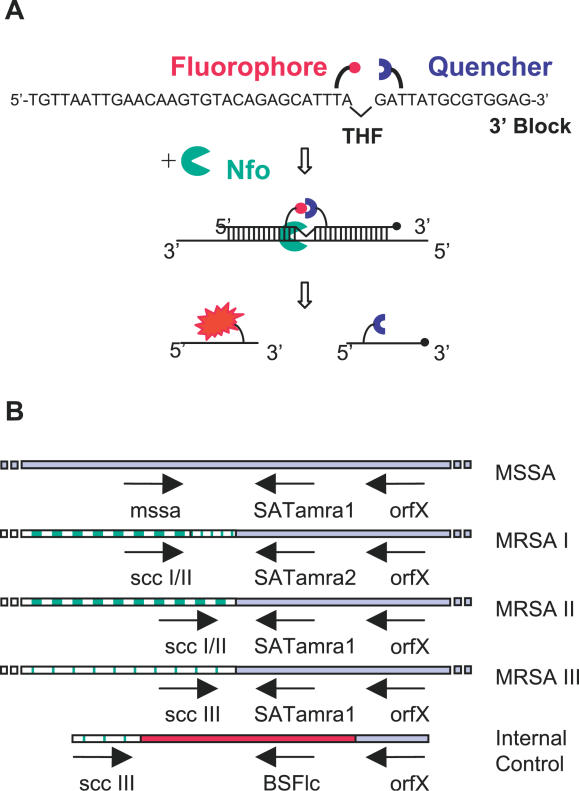
Schematic of the Probe-Based RPA Detection Method (A) Signal generation by separation of a fluorophore and a quencher depends on cutting of the probe by double-strand specific Nfo. (B) Arrangement of primers and probes relative to the targets used in Figures 4 and 5. See Protocol S1 for sequences of MRSA isoforms. A PCR fragment that fused an unrelated sequence to the target sites sccIII and orfX served as internal control.

To devise a highly sensitive RPA detection system that is not affected by primer artefacts, we developed a probe-based detection method ( Figure 3A). The probe we use contains a tetrahydrofuran abasic–site mimic (THF) [ 9], flanked in close proximity by nucleotides modified with a fluorophore and a quencher. The fluorescence of the intact construct is low. A block at the 3′-end prevents the oligonucleotide from acting as an amplification primer. Pairing of the probe to complementary DNA enables the recognition of the THF by the double-strand–specific Escherichia coli endonuclease IV (Nfo) [ 10]. The need for formation of a stable DNA duplex acts as an additional specificity-proofreading step in the context of our detection approach. Subsequent cutting of the probe separates the fluorophore/quencher complex and leads to a measurable increase in fluorescence. The cleavage reaction generates a free 3′ OH-end on the 5′ remnant of the incised probe. This oligomer can then be elongated by Bsu polymerase, thus serving as an amplification primer.

**Figure 4:**
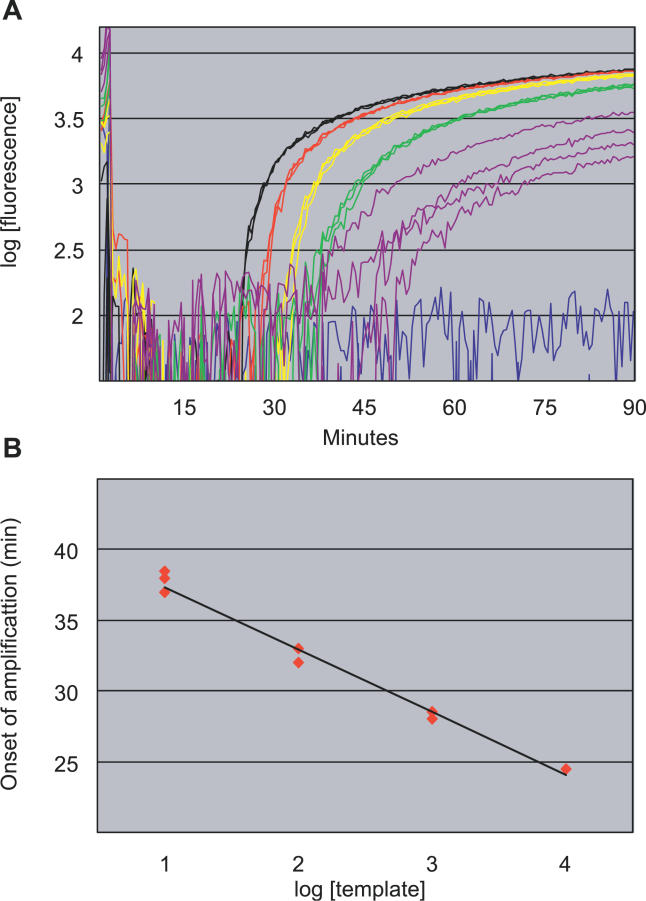
RPA Enables Specific DNA Amplification from Few Template Molecules (A) Probe signal of RPA reactions using the primer set orfX/sccIII (see Figure 3). MRSAIII DNA at 10 ^4^ (black, reactions 1–3), 10 ^3^ (red, 4–6), 100 (yellow, 7–9), ten (green, 10–12), or two (purple, 13–17) copies or water (blue, 18–20) served as template. (B) A plot of the onset time of amplification (defined as passing the 2.5 threshold) in reactions 1 to 12 in Figure 4A against the logarithm of the template copy number reveals a linear relationship.

**Figure 5:**
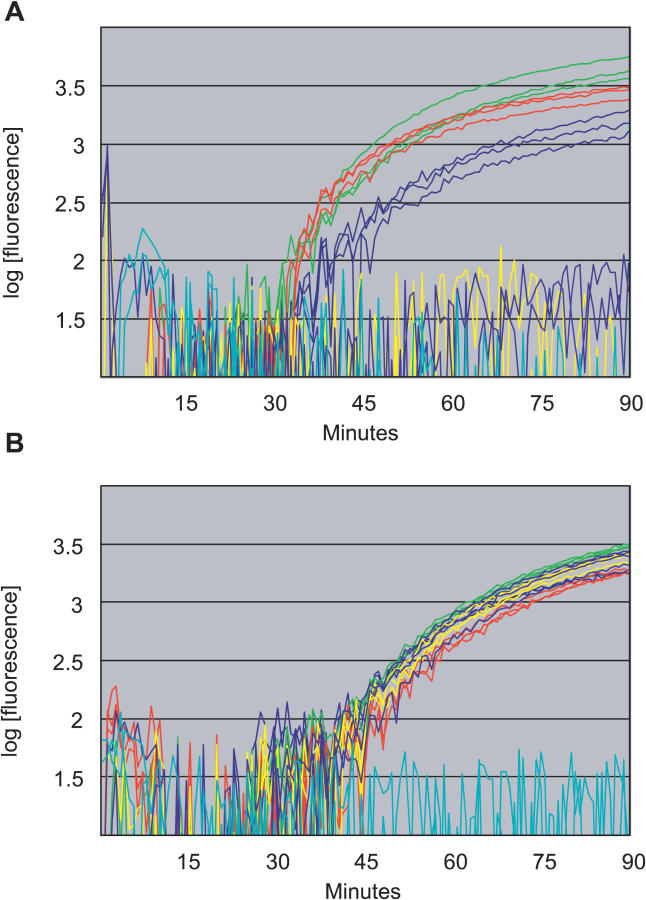
A Multiplex RPA Approach Enables Detection of Different MRSA Alleles and an Internal Control in the Same Reaction (A) MRSAI (green), MRSAII (dark blue), MRSAIII DNA (red) at ten copies, or MSSA DNA at 10 ^4^ copies (blue, negative control) or water (yellow, turquoise, negative controls) served as a template (in triplicate for each template condition). See Figure 3 for the arrangement of primers and probes used. (B) Detection of 50 copies of internal control DNA included in the reactions in (A). Note that two of the negative control sets (blue, yellow) included internal control template, whereas one set of reactions (turquoise) contained water only and served as “double negative” control.

To demonstrate the performance of combined RPA and probe/nuclease-based read-out, we designed primers and probes for the detection of the common hospital pathogen methicillin-resistant Staphylococcus aureus (MRSA; Figure 3B) [ 11]. The sensitivity and reproducibility of RPA were explored by the amplification of the staphylococcal cassette chromosome *mec* (SCC *mec*) integration locus of the isoform MRSAIII ( Figure 4A, see Protocol S1 for sequences). The onset of amplification at identical starting template quantities of ten, 100, 1,000, or 10,000 copies was virtually simultaneous, demonstrating the robustness of the technique. A plot of the time of beginning amplification against the logarithm of starting copy number reveals a linear relationship ( Figure 4B). At low template amounts (two copies), the amplification times are distributed over a wider period, with one reaction failing to generate a signal at all, and the correlation between onset of amplification and template concentration breaks down. However, product detection in samples with such low template concentrations supports the notion that RPA can essentially attain single template copy sensitivity.

The high sensitivity and specificity of RPA allow the design of a multiplex approach in which different amplicons are generated and detected in the same reaction. Three polymorphic alleles of the SCC *mec* integration region represent the vast majority of known MRSA genotypes (MRSAI through III; Figure 5A) [ 12]. While they share the genomic locus *orfX*, they differ in the sequence of the SCC *mec*. The integrating elements of MRSAI and II are homologous to each other except for a 102–base-pair insertion/deletion, while the MRSAIII SCC *mec* is highly divergent. A primer shared by all alleles was designed for the common region (orfX), whereas primers specific for MRSAI/II and MRSAIII target the different SCC *mec* variants (sccI/II and III, respectively). To ensure detection of all three isoforms, we designed two highly homologous probes (to account for polymorphisms) for the common region (SATamra1 and SATamra2). Fusing the target sequences for the primers sccIII and orfX to an unrelated DNA sequence created an internal control for the reaction. To verify the activity of each sample, the amplification of this construct was monitored simultaneously with that of the MRSA targets using another probe of appropriate sequence and bearing a different fluorophore/quencher pair (BSFlc). We employed this combination of primers and probes to detected ten genomic copies of the three MRSA types ( Figure 5B). By contrast, the closely related methicillin-sensitive Staphylococcus aureus (MSSA; 10 ^4^ copies) serving as a negative control did not generate a signal, despite being amplified by suitable primers (see Figure S5). The internal control was concomitantly detected in all samples.

**Figure 6:**
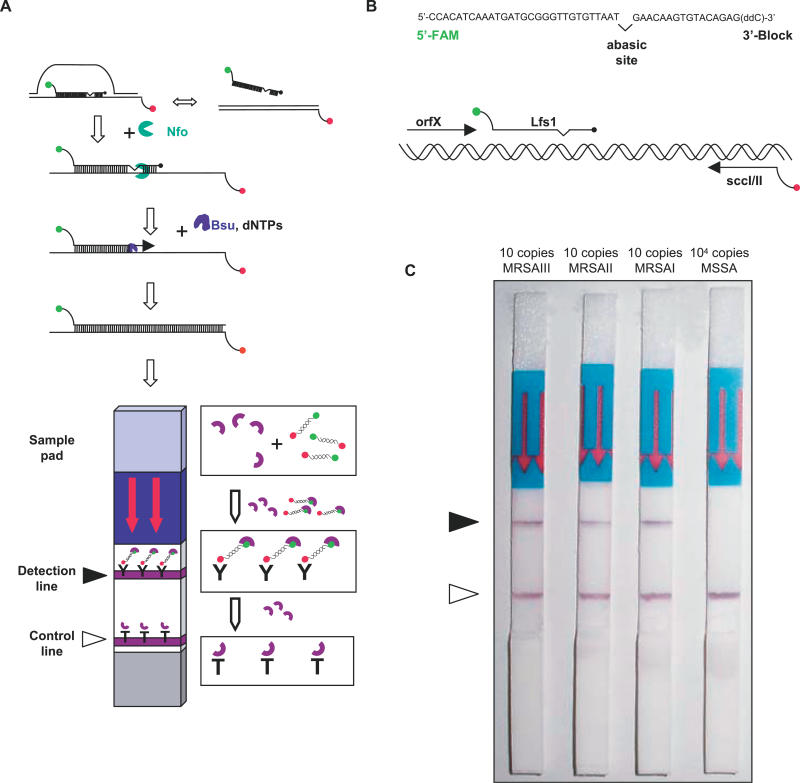
RPA for MRSA Detection Can Be Combined with an Instrumentation-Free Read-out System (A) Schematic of the principle of product generation and lateral-flow strip detection (see text for details). FAM label (green), biotin label (red), α-FAM-gold (purple), α-biotin antibody (Y, detection line, filled arrowhead), species-specific α-[α-FAM-gold] antibody (T, control line, open arrowhead). (B) Schematic of the lateral-flow probe design (top, here Lfs1) and its arrangement relative to amplification primers (bottom, here orfX and sccI/II). FAM label (green), biotin label (red). (C) Lateral-flow strips used for the detection of RPA products. Reactions contained (left to right) ten copies of MRSAIII, ten copies of MRSAII, ten copies of MRSAI, or 10,000 copies of MSSA (negative control) as template. Positive signals are generated in the first three reactions (filled arrowhead). The MSSA control reaction only produced a flow-control signal.

The combination of RPA and the described nuclease-sensitive fluorophore/quencher probes constitutes a DNA amplification/detection system that would require no other equipment than a simple handheld fluorometer, thus making quantitative DNA testing potentially accessible in a nonlaboratory point-of-care environment. We devised an even simpler detection approach by employing lateral-flow dipstick technology. This system is often used as a simple disposable diagnostic device. It employs pairs of specific antibodies to immobilise and detect entities containing two antigenic labels ( Figure 6A). Reaction mixtures (see below) are applied to a sample pad soaked in visible gold particles that are coupled to an antibody recognising one antigen. The complexes then travel in a buffer stream through the membrane and an additional, immobilised antibody captures the second antigen. If the antigens are conjoined in a DNA duplex, a coloured line appears at a defined location on the strip. A second line of immobilised antibodies captures the gold-conjugated antibody directly, whether conjoined to the other antigen or not, and serves as a flow control for the strip. In a variation of our probe detection system, we produced such dual antigen complexes by coupling biotin- and carboxyfluorescein (FAM)-bearing oligonucleotides in RPA amplicons ( Figure 6B). The 5′-biotinylated primer and its opposing counterpart ensure the efficient amplification of a target for probe binding. The probe, including a 5′-FAM label, an internal THF, and a 3′-blocking group, is incised by Nfo upon binding, creating a 3′ OH substrate for elongation by Bsu. The extension of the probe remnant stabilises its interaction with the biotin-labeled opposing strand and produces an amplicon that contains both antigens, biotin and FAM. The THF/3′ block prevents the production of biotin/FAM-containing primer artefacts, as processing of bona fide duplexes by Nfo adds a critical proofreading step. We used a multiplex approach similar to the one employed in Figure 5 to detect ten copies of each of the three MRSA isoforms and distinguish them from MSSA ( Figure 6C). As before, we designed two highly homologous probes (to account for polymorphisms) for the common region (Lfs1 and Lfs2). RPA reactions were followed by the application of the reaction mixtures to the sample pads of lateral-flow strips. Samples containing the MRSA template created biotin/FAM-amplicons and generated visible signals on the antibiotin detection line. In contrast, the MSSA-containing negative control failed to produce a conjoined complex and gave rise to the flow-control line only.

A number of research and clinical applications could benefit from employing RPA. Perhaps most obviously, the technology offers a significant breakthrough for the development of nonlaboratory devices. When integrated with handheld instruments or entirely equipment-free DNA detection systems, RPA will enable an easy-to-use testing system for a variety of pathogens as well as field kits for other applications.

## Materials and Methods

### Proteins and DNA.

Coding sequences for uvsx, uvsy, gp32, Bsu, and Nfo were amplified from genomic DNA (DSMZ, Braunschweig Germany), fused to hexahistidine-tags (N-terminal for uvsY, Bsu, and Nfo, C-terminal for uvsX and gp32), and cloned into suitable expression vectors. Overexpression and purification were done by standard protocols using nickel-NTA resin (Qiagen, Valencia, California, United States). Human genomic DNA was from Promega (Madison, Wisconsin, United States), B. subtilis DNA was from ATCC (American Type Culture Collection, Manassas, Virginia, United States), and S. aureus DNAs were a gift from Jodi Lindsay (University of London, London, England). S. aureus strains were EMRSA-3 (SCC *mec* type I; MRSAI), EMRSA-16 (MRSAII), EMRSA-1 (MRSAIII), and wild-type MSSA.

### RPA conditions.

Reactions were performed at 37 °C for 60 min or as indicated. Standard conditions were 50 mM Tris (pH 7.9), 100 mM potassium acetate, 14 mM magnesium acetate, 2 mM DTT, 5% Carbowax20M, 200 μM dNTPs, 3 mM ATP, 50 mM phosphocreatine, 100 ng/μl creatine kinase, and 30 ng/μl Bsu; in the multiplex experiment, Carbowax20M was at 5.5%. Recombinase protein concentrations were 900 ng/μl gp32, 120 ng/μl uxsX, and 30 ng/μl uvsY. Primers used for amplification of human targets were apoB4, apoB300, sry3, sry4, pbdg5, pbdg6 (300 nM each), for *B. subtilis,* bs1 and bs2 were used (300 nM each) in the experiment in Figure 2B. For MRSA amplification, primers used were 500 nM sccIII, 100 nM orfX ( Figure 4A) or 265 nM sccI/II, 265 nM sccIII, 70 nM orfX ( Figure 5) or 240 nM Bio-sccI/II, 240 nM Bio-sccIII, 120 nM orfX ( Figure 6). Reaction volumes were 20 μl, except for the experiment shown in Figure 2A (40 μl).

### Real-time monitoring.

Real-time RPA was performed in a plate-reader (Flx-800; BioTek, Winooski, Vermont, United States) in the presence of SybrGreenI (1:50,000; Molecular Probes, Eugene, Oregon, United States) or fluorophore/quencher probes (Eurogentec, San Diego, California, United States). Three probes were used:

SATamra1 5′-tgttaattgaacaagtgtacagagcatt(T)a(H)ga(q1)tatgcgtggag-biotin-3′

SATamra2 5′-tgttaattgagcaagtgtatagagcatt(T)a(H)ga(q2)tatgcgtggag-biotin-3′

BSFlc 5′-catgattggatgaataagctgcagc(F)g(H)t(q3)aaaggaaactta-biotin-3′

Here, (T) is dT-TAMRA, (F) is dT-fluorescein, (H) is THF, (q1) is dT-BHQ1, (q2) is dT-BHQ2, and (q3) is dT-DDQ1. Probes were used at 60 nM SATamra1 (MRSAIII experiment) or at 45 nM SATamra1, 45 nM SATamra2, 60 nM BSFlc (multiplex experiment). Nfo was used at 200 ng/μl. Excitation/detection was at 485/525 nm (SybrGreenI, BSFlc) or 530/575 nm (SATamra1/2). Measurements were taken every 30 or 45 s (multiplex experiment). Fluorescence probe data were normalised (subtraction of water control average) and preamplification baseline adjusted. The logarithm of the read-out was plotted against reaction time.

### Lateral-flow strip detection.

For lateral-flow strip experiments, two probes were used at 75 nM each:

Lfs1 5′-FAM-ccacatcaaatgatgcgggttgtgttaat(H)gaacaagtgtacagag-ddC-3′

Lfs2 5′-FAM-ccacatcaaatgatgcgggttgtgttaat(H)gagcaagtgtatagag-ddC-3′

The 5′-biotinylated forms of sccI/II and sccIII were utilised as primers. For each reaction (20 μl), 1 μl was diluted with 5 μl of running buffer (PBS/3% Tween) and applied directly to HybriDetect strips (Milenia Biotec, Bad Nauheim, Germany) according to the manufacturer's instructions.

## Supporting Information

Figure S1 Titrations of Reaction Components to Determine Conditions that Support RPA Polyacrylamide gel electrophoresis of RPA reactions using primers for the human Sry locus. Reactions were performed at 37 °C for 120 min and contained the primers sry3 and sry4 at 300 nM, 50 mM Tris (pH 8.4), 80 mM potassium acetate, 10 mM magnesium acetate, 2 mM DTT, 3 mM ATP, 200 μM dNTPs, 20 mM phosphocreatine, 100 ng/μl creatine kinase, 5% Carbowax20M, 600 ng/μl gp32, 200 ng/μl uvsX, 60 ng/μl uvsY, and 30 ng/μl Bsu, except when a given component was that under investigation. Optimal quantities of (A) gp32, (B) uvsY, (C) uvsX, (D) Carbowax20M, (E) ATP, and (F) Bsu for effective amplification of this particular target were determined. G) ADP-β-S and (H) ATP-γ-S inhibit the reactions. And 1,500 copies/μl of the human Y-chromosomal DNA served as template in 30-μl reactions (per sample the equivalent of 10-μl reaction volume was loaded on the gel).
(159 KB PDF)

Figure S2 Investigation of Product Size Limits of RPA Agarose gel electrophoresis of RPA reactions using primers for the human apoB locus. Primer apoB4 was combined with opposing primers capable of generating products of the indicated sizes. Reactions were performed at 37 °C for 120 min, and conditions used were 50 mM Tris (pH 8.4), 80 mM potassium acetate, 10 mM magnesium acetate, 2 mM DTT, 3 mM ATP, 200 μM dNTPs, 20 mM phosphocreatine, 100 ng/μl creatine kinase, 5% Carbowax20M, 600 ng/μl gp32, 125 ng/μl uvsX, 25 ng/μl uvsY, and 30 ng/μl Bsu. And 450 copies of human DNA were used as template in 30-μl reactions (per sample the equivalent of 10-μl reaction volume was loaded on the gel). Note that some hairpin-mediated product duplication occurred, converting some of the 300–base-pair amplicon to 2× and 3× unit length (*). RPA failed to produce amplicons of 1,500 base-pairs or more.
(70 KB PDF)

Figure S3 Investigation of the Minimum Oligonucleotides Size Necessary to Support RPA Polyacrylamide gel electrophoresis of RPA reactions using primers for the three independent loci in human genomic DNA (apoB, D18S51, Sry). Primers were 25, 28, or greater than 31 bases, as indicated. Reactions were performed at 37 °C for 120 min. Conditions used were 50 mM Tris/Cl (pH 8.4), 80 mM potassium acetate, 10 mM magnesium acetate, 2 mM DTT, 3 mM ATP, 200 μM dNTPs, 20 mM phosphocreatine, 100 ng/μl creatine kinase, 5% Carbowax20M, 600 ng/μl gp32, 200 ng/μl uvsX, 60 ng/μl uvsY, and 30 ng/μl Bsu polymerase. And 3,000 copies of target served as template in 30-μl reactions (per sample the equivalent of 10-μl reaction volume was loaded on the gel). The finding that a primer length of greater than 28 bases is required to support RPA is in good agreement with reports that investigated the ATP hydrolysis activity of uvsX-oligonucleotide filaments at different oligonucleotide sizes [ 3].
(17 KB PDF)

Figure S4 Restriction Digest of RPA Products Agarose gel electrophoresis of RPA reactions and their digestion products. Primers for the human loci apoB (primers apoB4, apoB300), Sry (primers sry3, sry4), and PBDG (primers pbdg5, pbdg6) were used. Reactions were performed at 37 °C for 60 min, and conditions used were 50 mM Tris (pH 7.9), 100 mM potassium acetate, 14 mM magnesium acetate, 2 mM DTT, 5% Carbowax20M, 200 μM dNTPs, 3 mM ATP, 300 nM each primer, 50 mM phosphocreatine, 100 ng/μl creatine kinase, and 30 ng/μl Bsu. Recombinase protein concentrations were 900 ng/μl gp32, 120 ng/μl uxsX, and 30 ng/μl uvsY. And 1,000 copies of human genomic DNA served as template. Reactions were performed in 40-μl volumes and purified using GenElute Clean-Up kits (Sigma, St. Louis, Missouri, United States). Ten percent of each reaction (equivalent to 4-μl RPA reaction) was either loaded directly (uncut) or after digestion with XbaI (apoB and Sry), HaeIII (apoB), or SmaI (PBDG) on a 2% agarose gel. The restriction pattern confirms the identity of the amplicons. Note that the amplicon generated by apoB4 and apoB300 includes an SNP covering the XbaI site. Since the template used is a combination of genomic DNA from several donors (Promega), the apoB RPA product consists of a mixture of fragments that either contain an XbaI site or are refractory to XbaI digestion.
(92 KB PDF)

Figure S5 Probe Signal of RPA Reactions Using the Primer Set orfX/MSSA and Probe SATamra2 (A) MSSA DNA at 10 ^4^ (black, reactions 1–3), 10 ^3^ (red, 4–6), 100 (yellow, 7–9), ten (green, 10–12), or two (purple, 13–17) copies or MRSAI DNA at 10 ^4^ copies (gray, reactions 18–20) or water (blue, 21–23) served as template. Reaction conditions were 50 mM Tris (pH 7.9), 100 mM potassium acetate, 14 mM magnesium acetate, 2 mM DTT, 200 μM dNTPs, 3 mM ATP, 20 mM phosphocreatine, 100 ng/μl creatine kinase, 5% Carbowax20M, 900 ng/μl gp32, 120 ng/μl uvsX, 30 ng/μl uvsY, and 30 ng/μl Bsu. Oligonucleotides were used at 500 nM MSSA, 100 nM orfX, and 60 nM SATamra2. While the MSSA target is amplified even at very low concentrations, the negative control (MRSAI) does not generate a signal.
(B) A plot of the onset time of amplification (defined as passing the 2.5 threshold) in reactions 1 to 12 against the logarithm of the template copy number reveals a linear relationship.
(53 KB PDF)

Protocol S1 Primer Sequences, Sequences of MRSA/MSSA Alleles, RPA Conditions, Primer Requirements, and Control DNA (32 KB DOC)

### Accession Numbers

The National Center for Biotechnology Information (NCBI) ( http://www.ncbi.nlm.nih.gov) accession numbers used in this paper are Bsu (NP_390787), T4 uvsX (NP_049656) T4 gp32 (NP_049854), T4 uvsY (NP_049799), apoB (AY324608), Sry (NM_003140), PBDG (AP003391), and Nfo (AP_002756) and SNP (SNP-ID rs693).

## Abbreviations

apoB: apolipoprotein B
Bsu: *Bacillus subtilis* Pol I
FAM: carboxyfluorescein
MRSA: methicillin-resistant *Staphylococcus aureus*
MSSA: methicillin-sensitive *Staphylococcus aureus*
PBDG: porphobilinogen deaminase
RPA: recombinase polymerase amplification
SCC *mec*: staphylococcal cassette chromosome *mec*
Sry: sex-determining region Y
THF: tetrahydrofuran abasic–site mimic
