# Supplementary material for: DNA Detection Using Recombination Proteins
Source: PLoS Biol. 2006 Jun 13;4(7):e204. doi: 10.1371/journal.pbio.0040204 (PMC1475771; doi:10.1371/journal.pbio.0040204)
Supplement: Supplementary file 1 [file pbio.0040204.sg001.pdf]

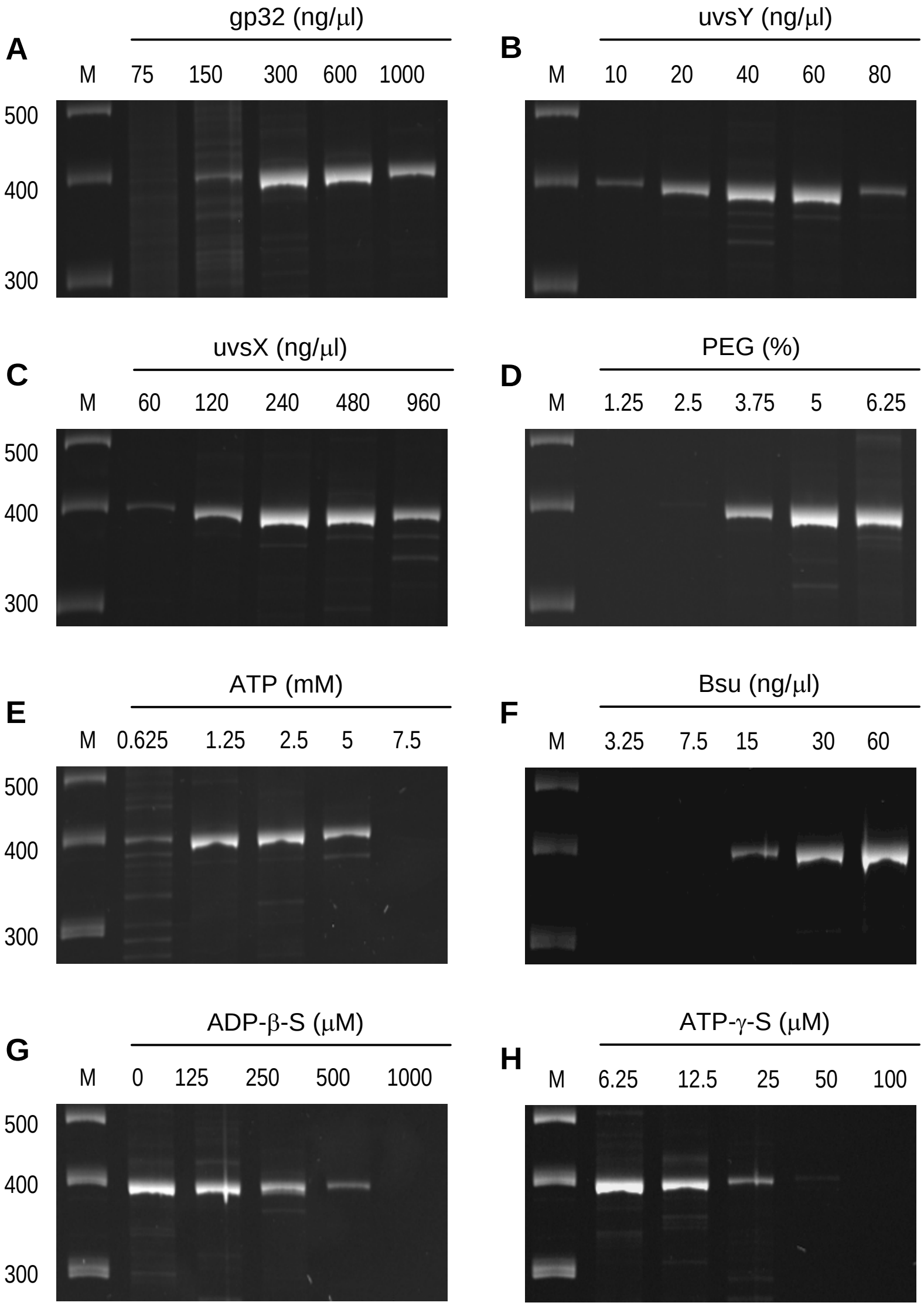
A
gp32 (ng/μl)
M 75 150 300 600 1000
500
400
300
B
uvsY (ng/μl)
M 10 20 40 60 80
C
uvsX (ng/μl)
M 60 120 240 480 960
500
400
300
D
PEG (%)
M 1.25 2.5 3.75 5 6.25
E
ATP (mM)
M 0.625 1.25 2.5 5 7.5
500
400
300
F
Bsu (ng/μl)
M 3.25 7.5 15 30 60
G
ADP-β-S (μM)
M 0 125 250 500 1000
500
400
300
H
ATP-γ-S (μM)
M 6.25 12.5 25 50 100
