# Supplementary material for: DNA Detection Using Recombination Proteins
Source: PLoS Biol. 2006 Jun 13;4(7):e204. doi: 10.1371/journal.pbio.0040204 (PMC1475771; doi:10.1371/journal.pbio.0040204)
Supplement: Supplementary file 2 [file pbio.0040204.sg002.pdf]

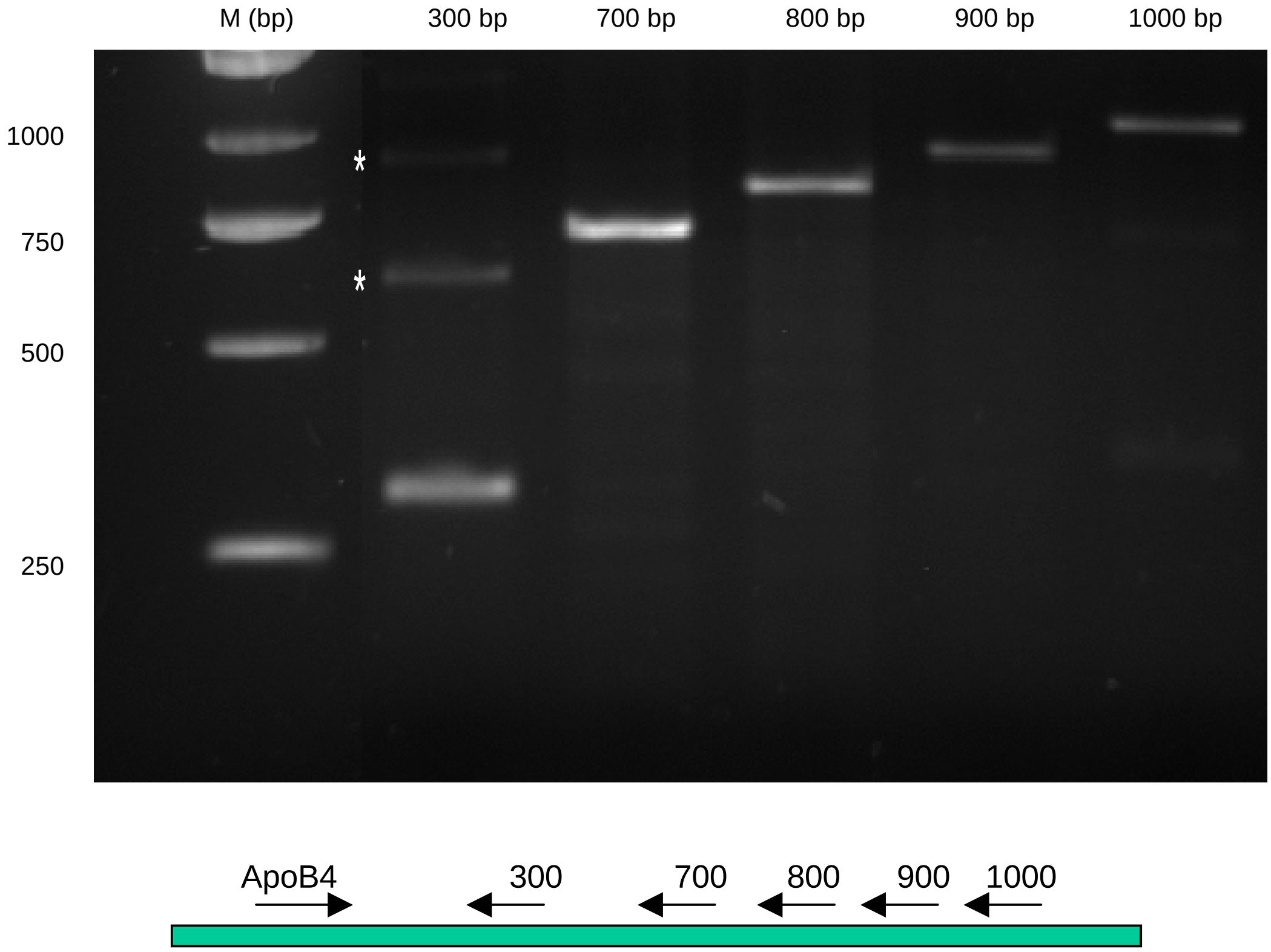

M (bp)
300 bp
700 bp
800 bp
900 bp
1000 bp
1000
750
500
250
*
*
ApoB4
300
700
800
900
1000
