# Supplementary material for: DNA Detection Using Recombination Proteins
Source: PLoS Biol. 2006 Jun 13;4(7):e204. doi: 10.1371/journal.pbio.0040204 (PMC1475771; doi:10.1371/journal.pbio.0040204)
Supplement: Supplementary file 3 [file pbio.0040204.sg003.pdf]

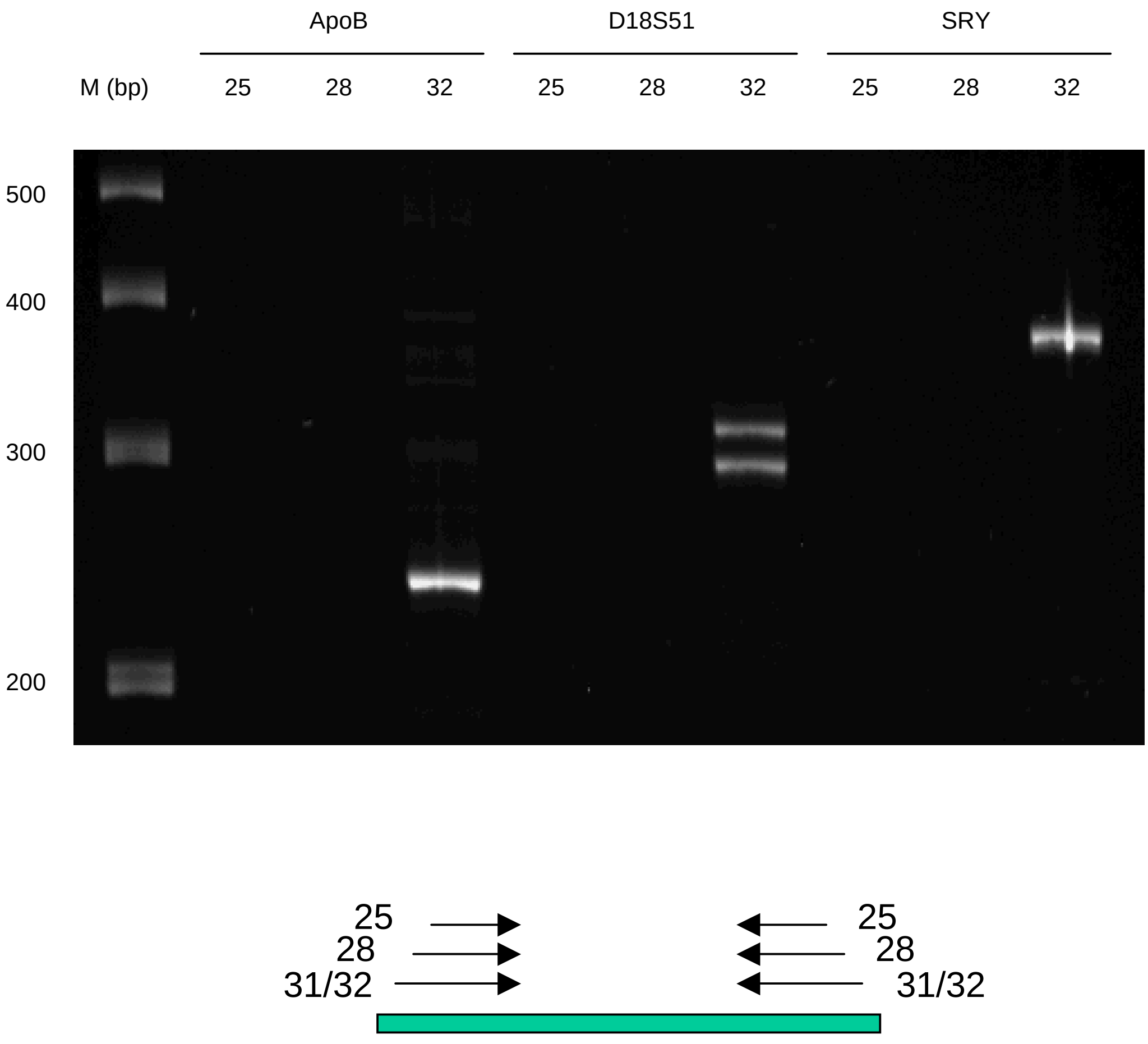
ApoB
D18S51
SRY
M (bp)
25
28
32
25
28
32
25
28
32
500
400
300
200
25
28
31/32
25
28
31/32
