# Supplementary material for: DNA Detection Using Recombination Proteins
Source: PLoS Biol. 2006 Jun 13;4(7):e204. doi: 10.1371/journal.pbio.0040204 (PMC1475771; doi:10.1371/journal.pbio.0040204)
Supplement: Supplementary file 4 [file pbio.0040204.sg004.pdf]

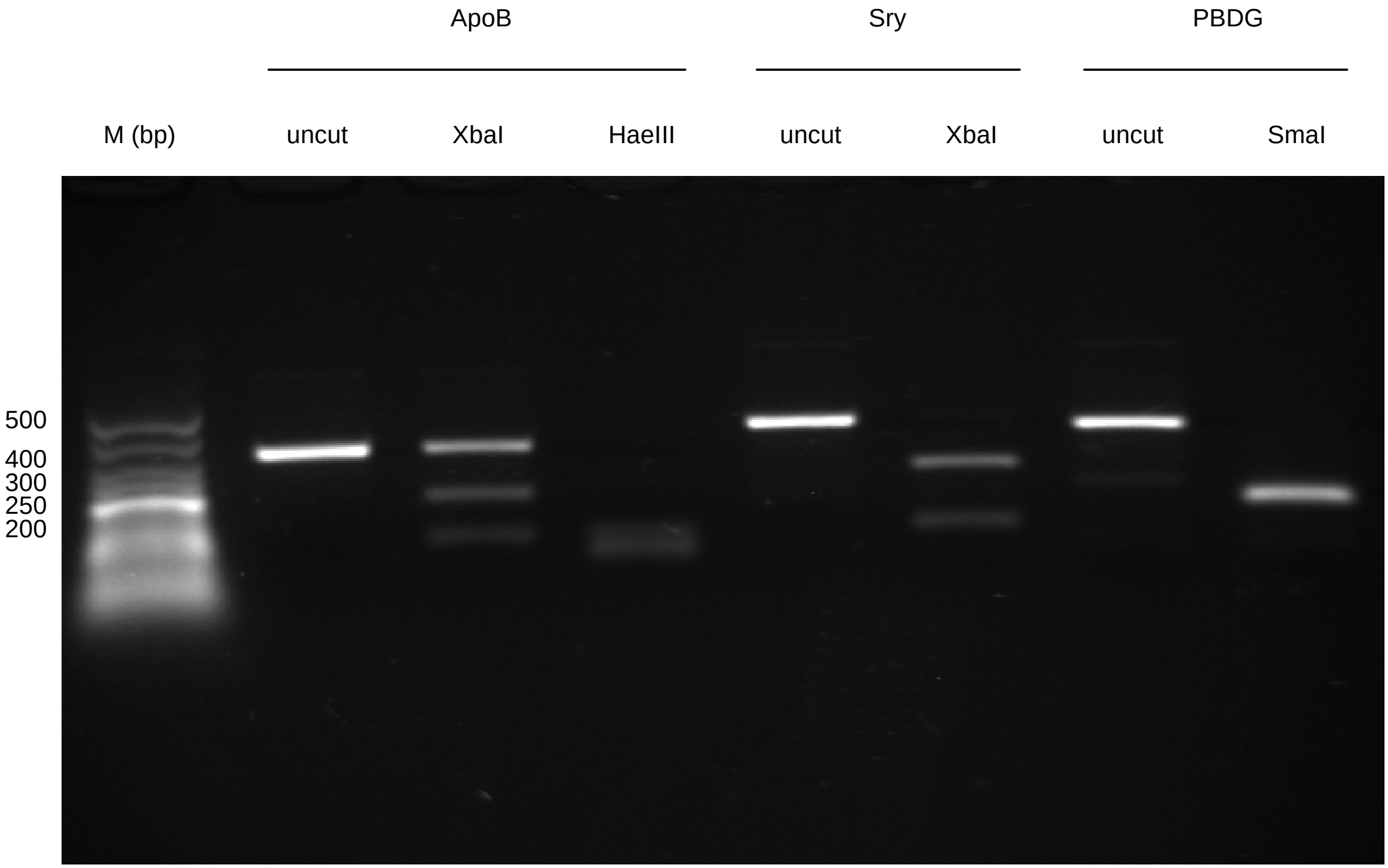

ApoB
Sry
PBDG
M (bp)
uncut
XbaI
HaeIII
uncut
XbaI
uncut
SmaI
500
400
300
250
200
