# Supplementary material for: DNA Detection Using Recombination Proteins
Source: PLoS Biol. 2006 Jun 13;4(7):e204. doi: 10.1371/journal.pbio.0040204 (PMC1475771; doi:10.1371/journal.pbio.0040204)
Supplement: Supplementary file 5 [file pbio.0040204.sg005.pdf]

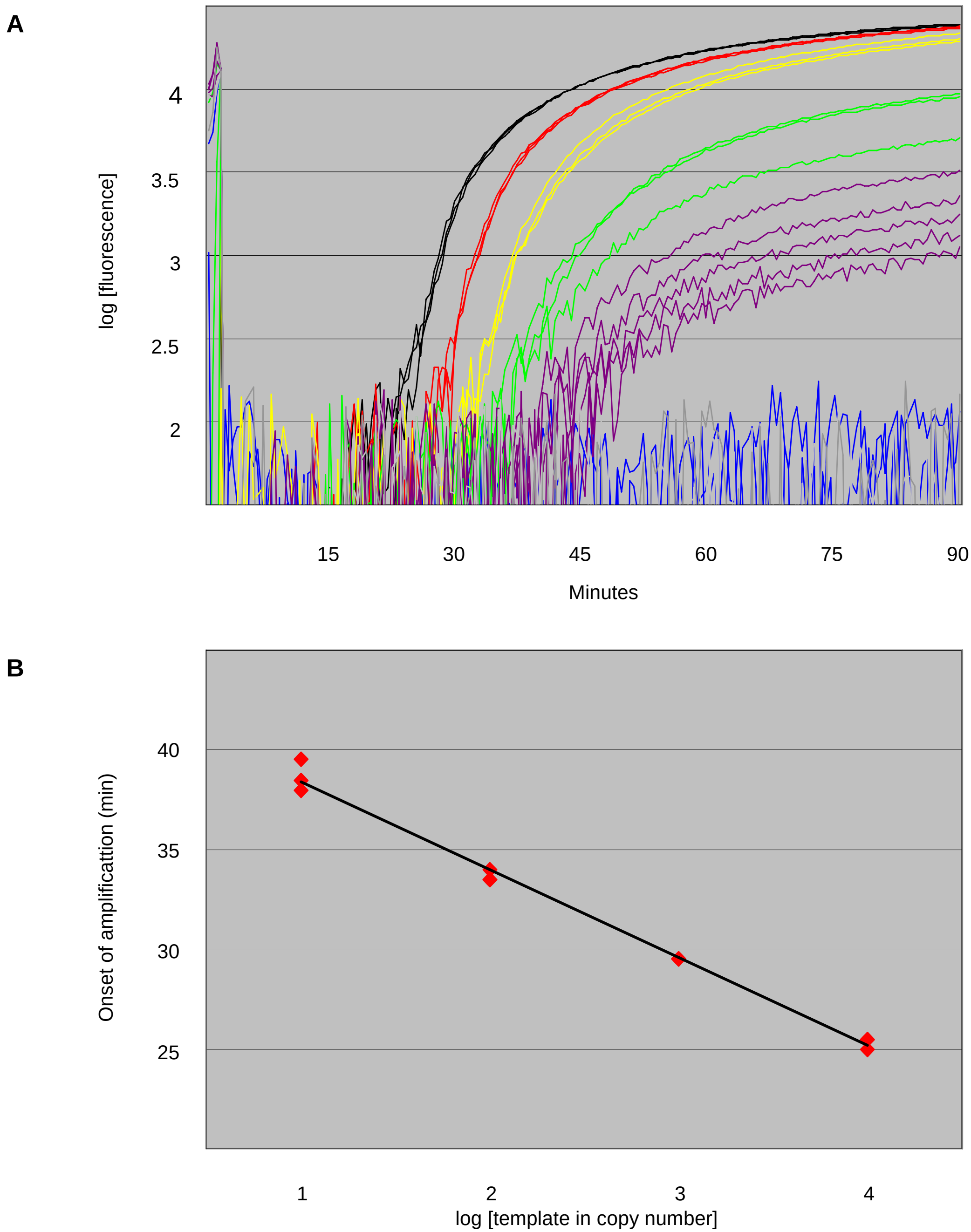
A
4
3.5
3
2.5
2
log [fluorescence]
15
30
45
60
75
90
Minutes
B
40
35
30
25
Onset of amplificattion (min)
1
2
3
4
log [template in copy number]
