# Supplementary material for: DNA Detection Using Recombination Proteins
Source: PLoS Biol. 2006 Jun 13;4(7):e204. doi: 10.1371/journal.pbio.0040204 (PMC1475771; doi:10.1371/journal.pbio.0040204)
Supplement: Supplementary file 6 [file pbio.0040204.sd001.doc]

# Protocol S1

# Primer sequences

Human loci ApoB, D18S51, Sry and PBDG:

#### apoB4 CAGTGTATCTGGAAAGCCTACAGGACACCAAAA

apoB300	TGCTTTCATACGTTTAGCCCAATCTTGGATAG

apoB700	TGGTAAACGGAAGTCTGGCAGGGTGATTCTCG

apoB800	CAATTGTGTGTGAGATGTGGGGAAGCTGGAAT

apoB900	GAGGTGGTTCCATTCCCTATGTCAGCATTTGC

apoB1000	GGGTTTGAGAGTTGTGCATTTGCTTGAAAATC

apoB500	atggtaaattctggtgtggaaaacctggatgg

apoB500-28	taaattctggtgtggaaaacctggatgg

apoB500-25	attctggtgtggaaaacctggatgg

apoB300rev	ctatccaagattgggctaaacgtatgaaagca

#### apoB300rev-28 ccaagattgggctaaacgtatgaaagca

apoB300rev-25	agattgggctaaacgtatgaaagca

D18S51 5'-28	gacatgttggcttctctctgttcttaac

D18S51 5'-25	atgttggcttctctctgttcttaac

D18S51 3'-28	gcacgtgcctgtagtctcagctacttgc

D18S51 3'-25	cgtgcctgtagtctcagctacttgc

sry3	aaagctgtaactctaagtatcagtgtgaaac

sry3-28	gctgtaactctaagtatcagtgtgaaac

sry3-25	gtaactctaagtatcagtgtgaaac

sry4	gttgtccagttgcacttcgctgcagagtacc

sry4-28	gtccagttgcacttcgctgcagagtacc

sry4-25	cagttgcacttcgctgcagagtacc

pbdg5	aacagcccaaagatgagagtgattcgcgtgg

pbdg6	agtcctgcccttcccagccacggctcagac

*B. subtilis* locus SpoB:

#### bs1 acggcattaacaaacgaactgattcatctgcttgg

bs2	ccttaatttctccgagaacttcatattcaagcgtc

#### **Sequences of MRSA/MSSA alleles**

MRSA/MSSA primers:

sccI/II	CTCAAAGCTAGAACTTTGCTTCACTATAAGTATTC

sccIII	CCAATATTTCATATATGTAATTCCTCCACATCTCA

orfX	CCCAAGGGCAAAGCGACTTTGTATTCGTCATTGGCGGATCAAACG

mssa	CCAATTTGATAGGGCCTAATTTCAACTGTTAGCTA

Genomic target sequences (primer target sites are bold/underlined, probe binding sites are in bold/italic):

MSSA

TTTTAGATATAAA**CCAATTTGATAGGGCCTAATTTCAACTGTTAGCTA**CTACTTAAGTTATATGCGCAATTATCGTGATATATCTTATATATTGAATGAACGTGGATTTAATGTCCACCATTTAACACCCTCCAAATTATTATCTCCTCATACAGAATTTTTTAGTTTTACTTATGATACGCCT***CTCCACGCATAATCTTAAATGCTCTATACACTTGCTCAATTAACA***CAACCCGCATCATTTGATGTGGGAATGTCATTTTGCTGAATGATAGTGCGTAGTTACTGCGTTGTAAGACGTCCTTGTGCAGGC**CGTTTGATCCGCCAATGACGAATACAAAGTCGCTTTGCCCTTGGG**TCATGCG

MRSAI:

TTTAGTTGCAGAAAGAATTTT**CTCAAAGCTAGAACTTTGCTTCACTATAAGTATTC**AGTATAAAGAATATTTCGCTATTATTTACTTGAAATGAAAGACTGCGGAGGCTAACTATGTCAAAAATCATGAACCTCATTACTTATGATAAGCTT***CTCCACGCATAATCTTAAATGCTCTATACACTTGCTCAATTAACA***CAACCCGCATCATTTGATGTGGGAATGTCATTTTGCTGAATGATAGTGCGTAGTTACTGCGTTGTAAGACGTCCTTGTGCAGGC**CGTTTGATCCGCCAATGACGAATACAAAGTCGCTTTGCCCTTGGG**TCATGCG

MRSAII:

TTTAGTTGCAGAAAGAATTTT**CTCAAAGCTAGAACTTTGCTTCACTATAAGTATTC**AGTATAAAGAATATTTCGCTATTATTTACTTGAAATGAAAGACTGCGGAGGCTAACTATGTCAAAAATCATGAACCTCATTACTTATGATAAGCTTCTTAAAAACATAACAGCAATTCACATAAACCTCATATGTTCTGATACATTCAAAATCCCTTTATGAAGCGGCTGAAAAAACCGCATCATTTATGATATGCTT***CTCCACGCATAATCTTAAATGCTCTGTACACTTGTTCAATTAACA***CAACCCGCATCATTTGATGTGGGAATGTCATTTTGCTGAATGATAGTGCGTAGTTACTGCGTTGTAAGACGTCCTTGTGCAGGC**CGTTTGATCCGCCAATGACGAATACAAAGTCGCTTTGCCCTTGGG**TCATGCG

MRSAIII:

AAGGTATAAT**CCAATATTTCATATATGTAATTCCTCCACATCTCA**TTAAATTTTTAAATTATACACAACCTAATTTTTAGTTTTATTTATGATACGCTT***CTCCACGCATAATCTTAAATGCTCTGTACACTTGTTCAATTAACA***CAACCCGCATCATTTGATGTGGGAATGTCATTTTGCTGAATGATAGTGCGTAGTTACTGCGTTGTAAGACGTCCTTGTGCAGGC**CGTTTGATCCGCCAATGACGAATACAAAGTCGCTTTGCCCTTGGG**TCATGCG

## RPA conditions

RPA relies on the establishment of a reaction environment that support the formation of recombinase-oligonucleotide complexes. Since the process is also ATP-dependent [3], it requires an energy regeneration system for sustained activity. We titrated key components of the RPA reaction mixture in order to determine their influence on amplification performance (Fig. S1). RPA proved to work robustly over a relatively wide range of reagent concentrations. We found, however, that optimal reaction conditions varied between different primer pairs and therefore had to be defined individually.

## Primer requirements

We used RPA to amplify of a wide range of targets. While the design of primers revealed no limitations on sequence composition itself, certain parameters have to be met for an oligonucleotide to be suitable for RPA. Amplicon size under the conditions employed is limited to approximately 1kb (Fig. S2). The minimum length of a primer proved to be about 30 nucleotides (Fig. S3). We observed variability in the performance of oligonucleotides that differ in sequences but are similar in length and position relative to their counterpart. The rules governing the influence of nucleotide sequence on the quality of a particular RPA primer are currently under investigation.

### Control DNA

# The wild-type *S. aureus* DNA (MSSA) [11,12] serving as a negative control in the experiment shown in 2C does act as a template for RPA when combined with the primer pair orfX/mssa (Fig. S5).
